# Agomelatine might be more appropriate for elderly, depressed, type 2 diabetes mellitus patients than paroxetine/fluoxetine

**DOI:** 10.18632/aging.203586

**Published:** 2021-10-05

**Authors:** Zihong Liang, Yanbo Jia, Lizhen Zhao, Runxiu Zhu, Xuemei He, Bagen Tong, Fan Yang, Lixia Hao, Pengfei Cui, Jun Yuan

**Affiliations:** 1 Department of Psychiatry, Inner Mongolia Autonomous Region People’s Hospital, Huhhot, Inner Mongolia 010017, People’s Republic of China; 2 Department of Orthopaedics, The Second Affiliated Hospital of Inner Mongolia Medical University, Huhhot, Inner Mongolia 010030, People’s Republic of China; 3 Department of Neurology, Inner Mongolia Autonomous Region People’s Hospital, Huhhot, Inner Mongolia 010017, People’s Republic of China; 4 Department of Psychiatry, Inner Mongolia Autonomous Region Third Hospital, Huhhot, Inner Mongolia 010050, People’s Republic of China

**Keywords:** type 2 diabetes mellitus, depression, agomelatine, paroxetine, fluoxetine

## Abstract

Agomelatine was a novel and melatonergic antidepressant. The present study was conducted to find out whether age was an important factor for agomelatine in treating depressed type 2 diabetes mellitus (T2DM) patients. In total, 193 depressed T2DM patients were included. There were 84 patients ranged from 27 years old to 49 years old (age phase I) (*n* = 44 receiving agomelatine, *n* = 40 receiving paroxetine or fluoxetine), and 109 patients ranged from 50 years old to 70 years old (age phase II) (*n* = 56 receiving agomelatine, *n* = 53 receiving paroxetine or fluoxetine). The Hamilton Depression Rating Scale (HDRS) score, Hamilton Anxiety Rating Scale (HARS) score, fasting plasma glucose (FPG), hemoglobin A1c (HbA1c) level and body mass index (BMI) were assessed after 12 weeks treatment. After treatment, we found that among patients in age phase I, there were no significant differences in final average HDRS score, HARS score, FPG, HbA1c level, BMI, response rate and remission rate between the two groups. However, among patients in age phase II, compared to patients receiving paroxetine or fluoxetine, patients receiving agomelatine had the significantly lower average HDRS score, HARS score, HbA1c level and BMI, and significantly higher response rate and remission rate. The incidence of treatment-related adverse events was similar between the two groups in both age phases. These results suggested that age was an important factor for agomelatine in treating depressed T2DM patients. Compared to paroxetine/fluoxetine, agomelatine might be more appropriate for elderly depressed T2DM patients.

## INTRODUCTION

Diabetes mellitus (DM) is a common chronic and progressive metabolic disease characterized by notably abnormal glucose metabolism [1–4]. It can cause huge economic burden to individuals and society. Based on World Health Organization (WHO) estimation, the global prevalence of this disease is rising annually, up to about 450 million in 2019 [5]. In China, the number of DM patients is about 116.4 million in 2019, which is the largest such population worldwide [6]. There are two major types of DM: Type 1 and type 2 diabetes; the later accounts for 90–95% of those with DM [7]. It is estimated that there are a total of 1.6 million DM-related deaths in 2016 [6], and the number is increased to 4 million in 2019 [8].

In addition to early mortality or physical impairments, DM can also negatively impact the mental health of patients. Previous studies found that depression was more frequently diagnosed in DM patients compared to the matched healthy controls; about 15% of DM patients met the criteria for comorbid depression [9, 10]. Depression is a seriously debilitating mental illness with unclear pathogenesis and no objective diagnostic methods [11–13]. The depressed DM patients often show some negative coping strategies that can affect the outcomes of treatment, resulting in the decreased treatment adherence, increased diabetes fatalism and low-quality of life [14]. Therefore, it is important to treat depression in the management of DM.

The efficacy and safety of medications were the two important aspects in clinical application, and researchers have done many works to increase the efficacy and safety of medications [15–17]. Clinicians should be very careful in choosing antidepressants for DM patients, as some medications might be inappropriate for treating depression in DM patients. Clinicians should comprehensively assess the effects of antidepressants on glycemic control, especially in elderly DM patients [18, 19]. Previous studies showed that serotonin reuptake inhibitors (SSRIs) were appropriate for treating depression in DM patients [20, 21]. Fluoxetine was found to be effective in treating depressive symptoms and decreasing blood glucose levels in DM patients [20]. Paile-Hyvärinen et al. reported that paroxetine could effectively mitigate the depressive symptoms and improve glycemic control in DM patients [21]. Recently, a new antidepressant, agomelatine was reported to offer some advantages over SSRIs, such as paroxetine and fluoxetine, in treating the depressed DM patients [22, 23]. However, these studies did not take age into consideration when they obtained such conclusions. Thus, we conducted this study to investigate whether age was an important factor for agomelatine in treating depressed T2DM patients.

## RESULTS

### Depressive and anxiety symptoms assessment in age phase I

**Figure 1:**
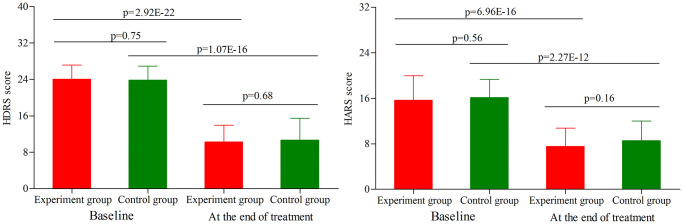
**HDRS score and HARS score in the two groups (age phase I) before and after treatment.** Abbreviations: HDRS: Hamilton Depression Rating Scale; HARS: Hamilton Anxiety Rating Scale.

As shown in Figure 1, the initial average HDRS score was similar between the two groups (*p* = 0.75). After treatment, the average HDRS score was significantly decreased in both groups (experiment group, *p* = 2.92E-22; control group, *p* = 1.07E-16). The final average HDRS score was still similar between the two groups (*p* = 0.68). Meanwhile, the two groups had the similar response rates (43.2% for experiment group and 50.0% for control group, *p* = 0.53) and remission rates (29.5% for experiment group and 37.5% for control group, *p* = 0.44). In addition, the initial average HARS score was similar between the two groups (*p* = 0.56) (Figure 1). After treatment, the average HARS score was significantly decreased in both groups (experiment group, *p* = 6.96E-16; control group, *p* = 2.27E-12). The final average HARS score was still similar between the two groups (*p* = 0.16).

### Depressive and anxiety symptoms assessment in age phase II

**Figure 2:**
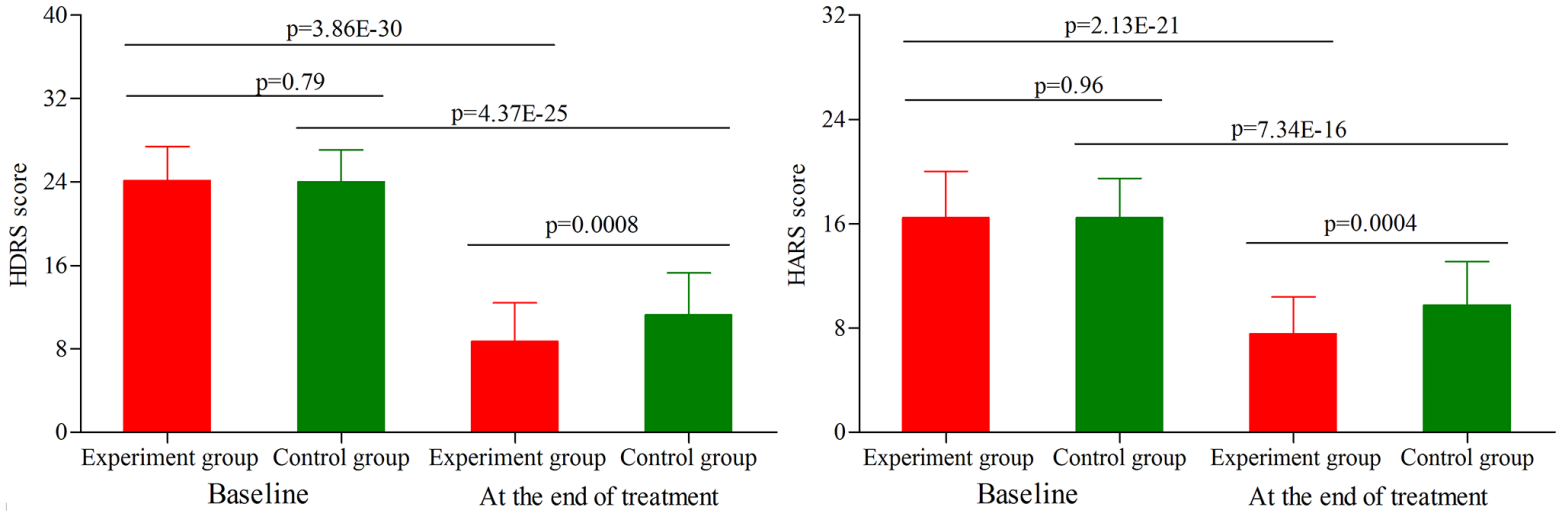
**HDRS score and HARS score in the two groups (age phase II) before and after treatment.** Abbreviations: HDRS: Hamilton Depression Rating Scale; HARS: Hamilton Anxiety Rating Scale.

As shown in Figure 2, the initial average HDRS score was similar between the two groups (*p* = 0.79). After treatment, the average HDRS score was significantly decreased in both groups (experiment group, *p* = 3.86E-30; control group, *p* = 4.37E-25). The final average HDRS score was significantly lower in the experiment group than in the control group (*p* = 0.0008). Meanwhile, we found that compared to the control group, the experiment group had the significantly higher response rate (52.8% vs. 73.2%, *p* = 0.03) and remission rate (24.5% vs. 42.9%, *p* = 0.04). In addition, the initial average HARS score was similar between the two groups (*p* = 0.96) (Figure 2). After treatment, the average HARS score was significantly decreased in both groups (experiment group, *p* = 2.13E-21; control group, *p* = 7.34E-16). The final average HARS score was significantly lower in the experiment group than in the control group (*p* = 0.0004).

### Metabolic control assessment in age phase I

**Figure 3:**
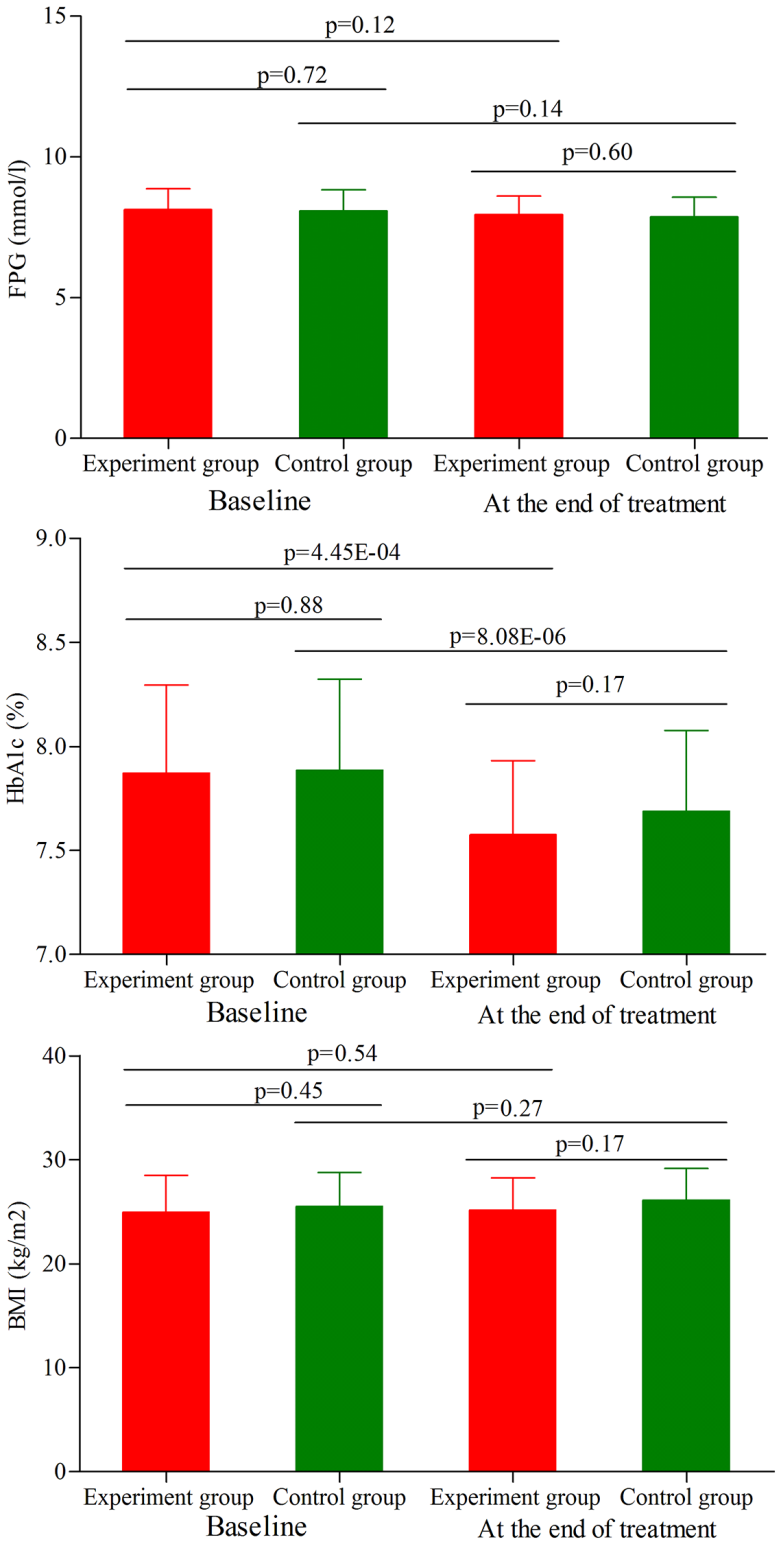
**FPG level, HbA1c level and BMI in the two groups (age phase I) before and after treatment.** Abbreviations: HbA1c: hemoglobin A1c; FPG: fasting plasma glucose; BMI: body mass index.

As shown in Figure 3, the initial average FPG level, HbA1c level and BMI were similar between the two groups (*p* = 0.72, *p* = 0.88, *p* = 0.45, respectively). At the end of treatment, the average FPG level was not significantly changed in both groups (Figure 3). Meanwhile, the results showed that the main effect of treatment on the final FPG level was not significant (*p* = 0.60), with similar final average FPG level between the two groups. Meanwhile, we found that the average HbA1c level was significantly decreased in both groups (experiment group, *p* = 4.45E-04; control group, *p* = 8.08E-06) (Figure 3). But the final average HbA1c level was sill similar between the two groups (*p* = 0.17). In addition, the average BMI was not significantly changed in both groups (Figure 3), and there was still no significant difference on the final average BMI between the two groups (*p* = 0.17).

### Metabolic control assessment in age phase II

**Figure 4:**
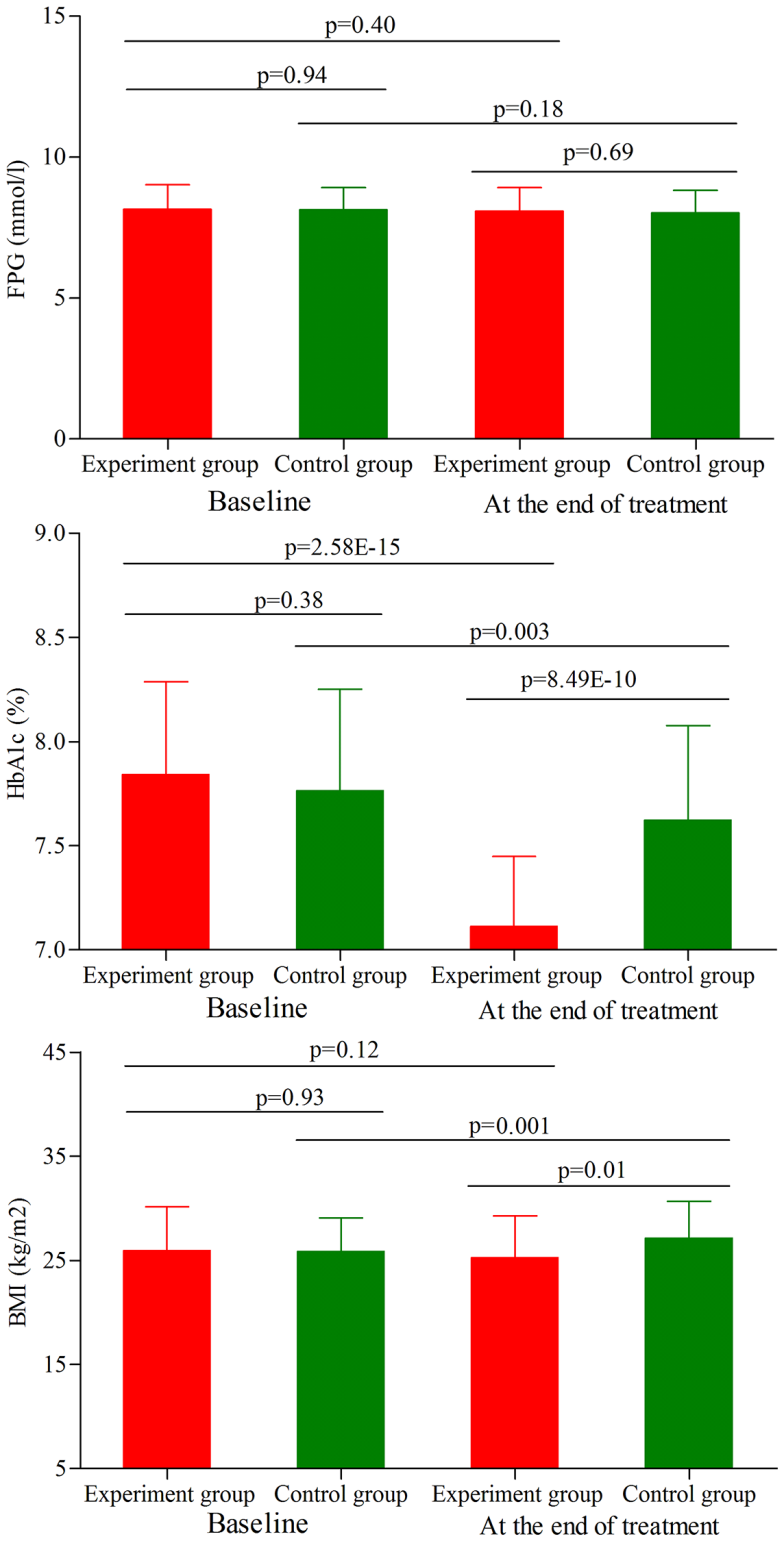
**FPG level, HbA1c level and BMI in the two groups (age phase II) before and after treatment.** Abbreviations: HbA1c: hemoglobin A1c; FPG: fasting plasma glucose; BMI: body mass index.

As shown in Figure 4, the initial average FPG level, HbA1c level and BMI were similar between the two groups (*p* = 0.94, *p* = 0.38, *p* = 0.93, respectively). At the end of treatment, the average FPG level was not significantly changed in both groups (Figure 4). Meanwhile, the results showed that the main effect of treatment on the final FPG level was not significant (*p* = 0.69), with similar final average FPG level between the two groups. Meanwhile, we found that the average HbA1c level was significantly decreased in both groups (experiment group, *p* = 2.58E-15; control group, *p* = 0.003) (Figure 4). But the final average HbA1c level was significantly lower in the experiment group than in the control group (*p* = 8.50E-10). In addition, we found that the average BMI was significantly increased in the control group after treatment (*p* = 0.001) (Figure 4), and there was significant difference on the final average BMI between the two groups (*p* = 0.01).

### Subgroup analysis on gender

**Figure 5:**
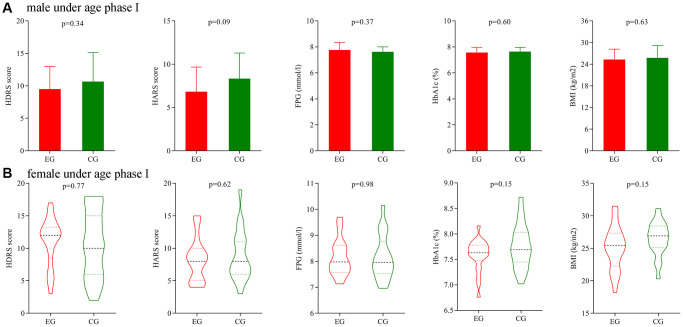
**Subgroup analysis on gender (age phase I).** (**A**) Final average HDRS score, HARS score, FPG level, HbA1c level and BMI in the two male groups. (**B**) Final average HDRS score, HARS score, FPG level, HbA1c level and BMI in the two female groups. Abbreviations: HDRS: Hamilton Depression Rating Scale; HARS: Hamilton Anxiety Rating Scale; HbA1c: hemoglobin A1c; FPG: fasting plasma glucose; BMI: body mass index; EG: experiment group; CG: control group.

Firstly, we analyze the potential gender differences using patients under age phase I. Before treatment, the initial average HDRS score, HARS score, FPG level, HbA1c level and BMI were not significantly different between the two male groups. After treatment, the final average HDRS score, HARS score, FPG level, HbA1c level and BMI were still not significantly different between the two male groups (Figure 5A). The similar results were found between the two female groups (Figure 5B).

**Figure 6:**
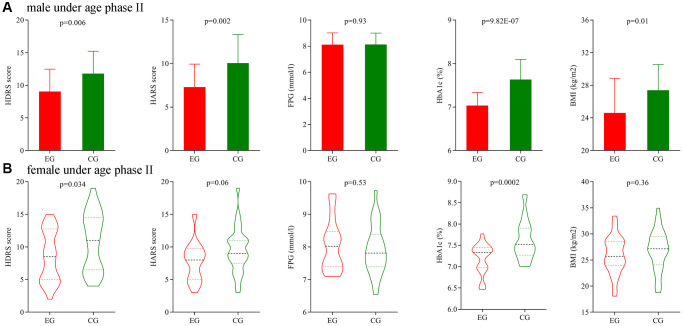
**Subgroup analysis on gender (age phase II).** (**A**) Final average HDRS score, HARS score, FPG level, HbA1c level and BMI in the two male groups. (**B**) Final average HDRS score, HARS score, FPG level, HbA1c level and BMI in the two female groups. Abbreviations: HDRS: Hamilton Depression Rating Scale; HARS: Hamilton Anxiety Rating Scale; HbA1c: hemoglobin A1c; FPG: fasting plasma glucose; BMI: body mass index; EG: experiment group; CG: control group.

Secondly, we analyze the potential gender differences using patients under age phase II. Before treatment, the initial average HDRS score, HARS score, FPG level, HbA1c level and BMI were not significantly different between the two male groups, and also not significantly different between the two female groups. After treatment, the final average HDRS score, HARS score, HbA1c level and BMI were significantly different between the two male groups (Figure 6A), and the final average HDRS score and HbA1c level were significantly different between the two female groups (Figure 6B).

### Subgroup analysis on depression severity

**Figure 7:**
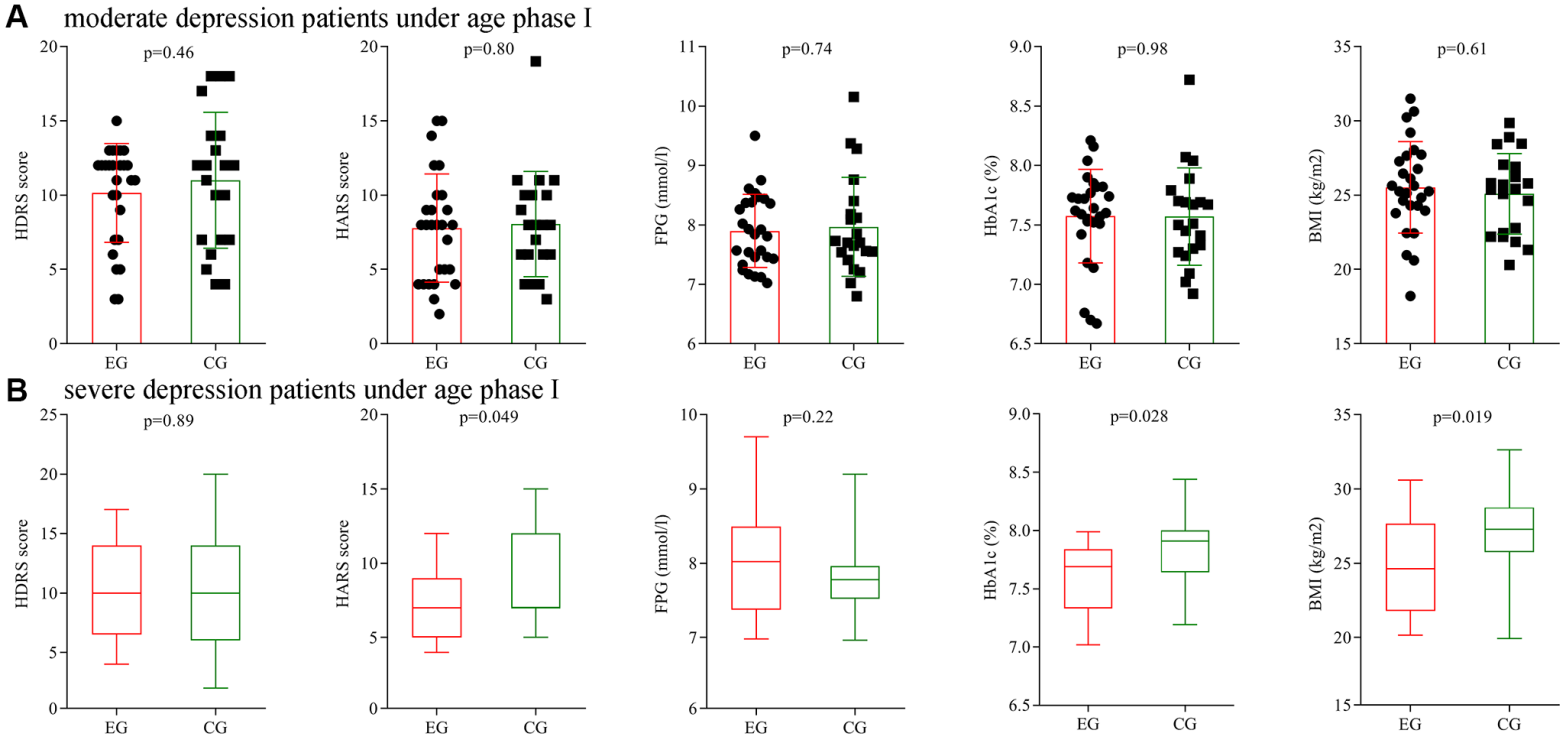
**Subgroup analysis on depression severity (age phase I).** (**A**) Final average HDRS score, HARS score, FPG level, HbA1c level and BMI in the two moderate depression groups. (**B**) Final average HDRS score, HARS score, FPG level, HbA1c level and BMI in the two severe depression groups. Abbreviations: HDRS: Hamilton Depression Rating Scale; HARS: Hamilton Anxiety Rating Scale; HbA1c: hemoglobin A1c; FPG: fasting plasma glucose; BMI: body mass index; EG: experiment group; CG: control group.

Firstly, subgroup analysis on depression severity was conducted using patients under age phase I. Before treatment, the initial average HDRS score, HARS score, FPG level, HbA1c level and BMI were not significantly different between the two moderate depression groups, and also not significantly different between the two severe depression groups. After treatment, the final average HDRS score, HARS score, FPG level, HbA1c level and BMI were still not significantly different between the two moderate depression groups (Figure 7A), but the final average HARS score, HbA1c level and BMI were significantly different between the two severe depression groups (Figure 7B).

**Figure 8:**
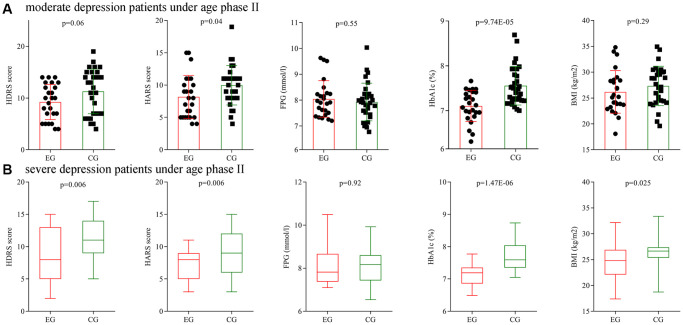
**Subgroup analysis on depression severity (age phase II).** (**A**) Final average HDRS score, HARS score, FPG level, HbA1c level and BMI in the two moderate depression groups. (**B**) Final average HDRS score, HARS score, FPG level, HbA1c level and BMI in the two severe depression groups. Abbreviations: HDRS: Hamilton Depression Rating Scale; HARS: Hamilton Anxiety Rating Scale; HbA1c: hemoglobin A1c; FPG: fasting plasma glucose; BMI: body mass index; EG: experiment group; CG: control group.

Secondly, subgroup analysis on depression severity was conducted using patients under age phase II. Before treatment, the initial average HDRS score, HARS score, FPG level, HbA1c level and BMI were not significantly different between the two moderate depression groups, and also not significantly different between the two severe depression groups. After treatment, the final average HARS score, HbA1c level and BMI were significantly different between the two moderate depression groups (Figure 8A), and the final average HDRS score, HARS score, HbA1c level and BMI were significantly different between the two severe depression groups (Figure 8B).

### Treatment-related adverse events

The used antidepressants in this study were well tolerated. These treatment-related adverse events were observed during the whole treatment period: nausea, headache, dry mouth, diarrhea, anxiety, dizziness, insomnia, suicidal intent, hyperhidrosis, vomiting, sexual side effects and anorexia. The incidence of treatment-related adverse events was not significantly different between the two groups (*p* = 0.54 in age phase I and *p* = 0.21 in age phase II).

## DISCUSSION

As far as we know, this was the first study to explore whether age was an important factor for agomelatine in treating depressed T2DM patients. At the end of treatment, both treatment methods could significantly decrease the HDRS score, HARS score and HbA1c level in both age phases. In age phase I, the deductions of these indexes were similar in both groups; but in age phase II, the deductions were significantly more in the experiment group than in the control group, and the experiment also had the significantly higher response rate and remission rate. The incidence of treatment-related adverse events was similar between the two groups in both age phases. In addition, in age phase II, compared to control groups, the experiment group had the significantly lower BMI after treatment. The subgroup analyses on gender and depression severity showed the similar results. Therefore, these results indicated that compared to paroxetine/fluoxetine, agomelatine might be more appropriated for depressed T2DM patients under age of 50–70 years old.

Age is a special and important factor in the lifetime of humans, which is characterized with inevitable and internal steady growth. In the different phases of life cycle (young, adult and old), individuals present different biological characteristics and disease risk [24, 25]. Previous studies have suggested that age could affect the microbial composition and functions in the mouse and cynomolgus macaques [26, 27]. Meanwhile, researchers also found the age-specific urinary metabolite signatures and gut microbiota composition in depressed patients [13, 28]. Thus, understanding the different characteristics in different age phases would be helpful for clinicians to prevent and treat diseases.

Depression and anxiety are two kinds of mental disorders, but they share a biological basis. Previous study found that neurotransmitters, such as serotonin and dopamine, played an important role in both depression and anxiety [29]. Depression and anxiety can co-occur, or appear sequentially (one in reaction to the other). Lamers et al. reported that of the patients with a primary anxiety disorder, more than 60% had a current or lifetime depression; similarly, of the patients with a primary depression, more than 65% had a current or lifetime anxiety [30]. Depression and anxiety may be not two kinds of disorders that coexist, but two faces of one. Therefore, to make our conclusion more robust and generalizable, the depression patients with anxiety disorder were also included in this study.

Previous studies found that depression might play a role in the pathogenesis of DM in some ways: i) some environmental stressors resulted in depression and simultaneously influenced the glucose metabolism [31, 32]; ii) depression could be an independent factor in influencing the nutrition and lifestyle behaviors of patients [31, 32]; iii) depression had a close relationship with the disturbed gut microbiota, and the stable gut microbiota was also important to the health of patients with DM [33–35]; iv) depression could lead to the over-activation of hypothalamic-pituitary-adrenal (HPA) axis and inflammatory response to stress [36, 37]; and the inflammation had an important role in the onset of DM [38–41]. Here, we found that these antidepressants could significantly reduce the HDRS score and HbA1c level in depressed T2DM patients. Therefore, it may be helpful to use antidepressants to treat DM patients with depression.

A meta-analysis showed that the antidepressant modalities were favorable both for depression and diabetes-related parameters [42]. SSRIs are safer for use in depressed DM patients, because they have less antiadrenergic side effects and are free of quinidine-like action. Agomelatine, as a novel and melatonergic antidepressant, is marketed for treating depression. Many studies reported that agomelatine was at least as effective as other antidepressants, such as SSRIs, in treating patients with depression [43–45]. Moreover, compared to SSRIs, agomelatine causes less or no discontinuation syndrome and sexual side effects. Here, we found that agomelatine produced better efficacies in depression/anxiety symptoms and metabolic control for patients under the age of 50–70 years old. The clinical applicability of agomelatine in treating elderly depressed T2DM patients showed greater promise and should be further studied.

Several limitations should be noticed here. Firstly, the relatively small number of depressed T2DM patients might weak the robust of our results; then our conclusions were needed future studies to validate and support. Secondly, all the included patients were from China and with the same ethnicity, which might limit the generalizability of our findings. Thirdly, the treatment time (12 weeks) was relatively short; then it was unknown that whether our conclusions were appropriate for patients receiving long-term treatment. Fourthly, the information about whether or not the included patients received any non-psychiatric medications was not collected; thus, the interaction effects between non-psychiatric medications and the used antidepressants should be further explored. Fifthly, due to the very small samples of patients with worse glycemic control, we did not assess whether patients with worse glycemic control could fare differently with antidepressant treatment. Finally, only the patients with age < = 70 years old were included; then future studies were needed to find out whether our results were similar in patients with age >70 years old.

## CONCLUSIONS

In conclusion, the chronicity and high prevalence of depression in T2DM patients, as well as its unpleasant impact on the quality of life and medical outcomes, demonstrated the importance of evidence-based depression treatments. Here, we found that age was an important factor for agomelatine in treating depressed T2DM patients. The clinicians should take age into consideration when using antidepressants, such as agomelatine to treat depressed T2DM patients. Our findings will be helpful for clinicians to make an optimal treatment plan for depressed T2DM patients.

## MATERIALS AND METHODS

### Included the qualified T2DM patients

In total, 193 depressed T2DM patients were included, and the treatment methods were obtained from the previous studies [22, 23]. There were 84 patients ranged from 27 years old to 49 years old (age phase I) (*n* = 44 in the experiment group, *n* = 40 in the control group), and109 patients ranged from 50 years old to 70 years old (age phase II) (*n* = 56 in the experiment group, *n* = 53 in the control group). Patients in the experiment groups received agomelatine 25–50 mg/day, and patients in the control groups received paroxetine 20–40 mg/day or fluoxetine 30–40 mg/day. The doses of the used antidepressants remained unchanged during the whole treatment period. The separate computer-generated random number sequence was used to do randomization. The patients were under standard treatment for diabetes. These patients met the following criteria: i) hemoglobin A1c (HbA1c) >7.0% [46]; ii) 17-item Hamilton Depression Rating Scale (HDRS) was used here to assess the depression severity, and patients with HDRS score > = 17 were included; iii) patients were not currently under any psychoactive treatments; iv) without any other psychiatric disorders (not include anxiety disorder), suicidal ideation, serious mental or physical disease; and v) treatment was continued for 12 weeks. The detailed information of these included patients was described in Table 1.

**Table 1:** Demographics data of the included patients.

**Variables**	**Age phase I (age <50 years old)**		***P***	**Age phase II (50–70 years old)**		***P***
	**Agomelatine**	**Paroxetine/Fluoxetine**		**Agomelatine**	**Paroxetine/Fluoxetine**	
Number	44	40	−	56	53	−
Age (years)	41.14 (7.13)	42.65 (5.61)	0.29	57.59 (5.42)	58.88 (5.83)	0.24
Sex (F/M)	22/22	19/21	0.82	28/28	29/24	0.63
BMI (kg/m^2^)	24.93 (3.58)	25.50 (3.36)	0.45	25.94 (4.19)	25.88 (3.19)	0.93

### Outcomes measurement

To reduce the potential bias, the rater was blind to the drug regimen of patients. The HDRS score, Hamilton Anxiety Rating Scale (HARS) score, HbA1c value, fasting plasma glucose (FPG) and body mass index (BMI) were assessed before treatment and at the end of treatment. Response to drugs was defined as decrease in HDRS score of > = 50% from baseline, and remission was defined as a HDRS score of 7 or less. The acceptability of medications was assessed using the number of treatment-related adverse events. In addition, to assess whether our results were appropriate for patients with different genders or patients with different severity of depression, subgroup analyses on gender and severity of depression was conducted. Patients with HDRS value ranged from 17 to 24 were divided in moderate depression group, and patients with HDRS value greater than 24 were divided in severe depression group [47].

### Statistical analyses

SPSS statistics 19.0 was used to do all the statistical analyses. The mean and standard deviation were used to display the data characterized by a normal distribution. An independent Student’s *t*-test, paired *t*-test, chi-squared test or non-parametric test was conducted when appropriate. If the variables have normal distribution, the Student’s *t*-test was used; if not, the non-parametric test was used. When assessing the differences on final HDRS score, HARS score, HbA1c value, FPG value and BMI between the two groups, we used the analysis of covariance (ANCOVA) method [48]. This method could exclude the effect of parameter’s initial value when we examine the effect of treatment methods on the parameter’s final value. All tests were two-sided and a *p*-value <0.05 was considered as statistically significant.
